# A1E reduces stemness and self-renewal in HPV 16-positive cervical cancer stem cells

**DOI:** 10.1186/s12906-016-1013-4

**Published:** 2016-02-02

**Authors:** Taeho Kwon, Yesol Bak, Sun-Young Ham, Dae-Yeul Yu, Do-Young Yoon

**Affiliations:** 1 Department of Bioscience and Biotechnology, Bio/Molecular Informatics Center, Konkuk University, 1 Hwayang-dong, Gwangjin-gu, Seoul, 143-701 Republic of Korea; 2 Aging Intervention Research Center, Korea Research Institute of Bioscience and Biotechnology (KRIBB), Gwahak-ro 125, Yuseong-gu, Daejeon, 305-333 Republic of Korea

**Keywords:** Cervical cancer, Cancer stem cells, Self-renewal, EMT, Stemness

## Abstract

**Background:**

Cervical cancer is the second most common cancer in females. Recent reports have revealed the critical role of cervical cancer stem cells (CSCs) in tumorigenicity and metastasis. Previously we demonstrated that A1E exerts an anti-proliferative action, which inhibits the growth of cervical cancer cells.

**Methods:**

A1E is composed of 11 oriental medicinal herbs. Cervical cancer cell culture, wund healing and invasion assay, flow cytometry, sheroid formation assay, and wstern blot assays were performed in HPV 16-positive SiHa cell and HPV 16-negative C33A cells.

**Results:**

A1E targets the E6 and E7 oncogenes; thus, A1E significantly inhibited proliferation of human papilloma virus (HPV) 16-positive SiHa cells, it did not inhibit the proliferation of HPV-negative C33A cells. Accordingly, we investigated whether A1E can regulate epithelial-to-mesenchymal transition (EMT), CSC self-renewal, and stemness-related gene expression in cervical cancer cells. Down rgulation of cell migration, cell invasion, and EMT was observed in A1E-treated SiHa cells. Specifically, A1E-treated SiHa cells showed significant decreases in OCT-3/4 and Sox2 expression levels and in sphere formation. Moreover, CSCs makers ALDH+ and ALDH, CD133 double positive cell were significantly decreased in A1E-treated SiHa cells. However, A1E treatment did not down regulate ALDH+ expression and the number of ALDH/CD133 double positive cells in C33A cells.

**Conclusions:**

Taken together, A1E can inhibit CSCs and reduce the expression of stemness markers. Treating CSCs with A1E may be a potential therapy for cervical cancer.

## Background

Cervical cancer is the second most common cancer in women worldwide, despite many steps taken to reduce disease burden in past decades [1]. Infection with HPV subtypes such as HPV 16, 18, 31, and 33 greatly increases the risk of cancer and plays a central role in the development of ~99.5 % of cervical cancers [2]. The E6 and E7 oncoproteins have been shown to be main mediators of the development of HPV-induced cervical carcinoma [3]. The cancer stem cell (CSC) hypothesis states that a tumor has a hierarchical cellular structure in which only a small subpopulation, referred to as cancer stem cells, is capable of tumorigenesis [4]. CSCs possess stem cell-like properties of self-renewal and can differentiate into non-stem tumor cells. CSCs have been reported in multiple types of solid tumor and in cultured cancer cell lines, including brain, breast, colon prostate, and cervical cancer cell lines [5]. CSCs have been identified and characterized in cervical cancer cell lines, but there are hardly any reports of CSCs in samples from patients with cervical cancer [6]. ALDH, CD133, and Sox2 have been identified as the first putative CSC markers in human cervical carcinoma [6]. A study demonstrated the use of CD44 and cytokeratin 17 cell surface markers to enrich a cervical CSC population [3]. Further, another investigation revealed that an enriched cervical cancer cellular pool possesses tumorigenic capacity and expresses stemness-related genes (*Oct-3/4* and *Sox2*) [7]. Moreover, CSCs are responsible for cancer recurrence. Based on these reports, further cancer therapies might focus on the elimination of CSCs [8]. Moreover, epithelial-to-mesenchymal transition (EMT) is a major cause of CSC progression and differentiation [9]. Hence, development of innovative therapeutics is required. Herbal medicines have been used to treat various diseases since ancient times in many Asian countries. Herbal medicines are extracted from traditional Asian medical plants and have therapeutic activities against cancers, angiogenesis, and metastasis in the absence of observable in vivo side effects [10]. Previously we reported that A1E, an extract formulated from 11 Asian traditional medicinal plants, consists of several compounds that target pathways such as the E6 and E7 pathway and mitochondrial intrinsic pathway. A1E inhibits E6 and E7 oncogenes and induces intrinsic apoptosis via p53/pRb dependent pathways and a mitochondria-mediated pathway [11]. Furthermore, A1E induces apoptosis via activation of both extrinsic and intrinsic pathways and the inhibition of PI3K/Akt survival signaling pathways in lung cancer cells [12]. Based on these data, we investigated whether A1E can regulate EMT, CSC self-renewal, and stemness-related gene expression in cervical cancer cells. Our data suggest that A1E can inhibit CSCs and reduce the expression of stemness markers. Treating CSCs with A1E may be a potential therapy for cervical cancer.

## Methods

### A1E formulation

A1E is composed of 11 oriental medicinal herbs (Table 1). We previously determined proportions (w/w) of these herbs in A1E as follows [12] and used the same batch for analysis in this study: 15.8 % ginseng (Korea), 15.8 % Chaga (Russia), 10.6 % Pinellia tuber (China), 10.6 % Psoraleae semen (India), 5.2 % Alpinia rhizome (China), 5.2 % Sparganium rhizome (China), 5.2 % cinnamon bark (Vietnam), 5.2 % Astragalus root (Korea), 5.2 % Alpiniakatsumadai seed (Vietnam), 10.6 % Arisaema rhizome (China), and 10.6 % Dolichos seed (China). The herbal ingredients were obtained from Oriental Medical Hospital of Dongkuk University Ilsan, Korea) and kindly authenticated by Dr. Jeong Seong hyun (Department of Oriental Herbal Materials, Dongkuk University). Extraction of A1E and constituent herbs. The ethanol extract was prepared as follows: The dried and pulverized medicinal herbs were mixed together and 1 Kg batch was soaked with 40 % ethanol (3 liters) the ethanol extract was concentrated with a rotary evaporator, and lyophilized, and reconstituted in either dimethysulfoxide (DMSO) for the in vitro studies. Additional procedures were performed as described previously [12].

**Table 1:** The composition of A1E

Oriental name	Country of origin	Grams	%
Ginseng	Korea	158	15.8
Chaga	Russia	158	15.8
Pinellia tuber	China	106	10.6
Psoraleae semen	India	106	10.6
Alpinia rhizome	China	52	5.2
Sparganium rhizome	China	52	5.2
Cinnamon bark	Vietnam	52	5.2
Astragalus root	Korea	52	5.2
Alpiniakatsumadai seed	Vietnam	52	5.2
Arisaema rhizome	China	106	10.6
Dolichos seed	China	106	10.6
Total amount		1000	100

### Cell culture

The HPV 16-positive SiHa and the HPV-negative C33A cervical cancer cell lines were obtained from the American Type Culture Collection (USA). The cells were maintained in Dulbecco’s modified Eagle’s medium (DMEM, Invitrogen) containing 10 % fetal bovine serum (FBS, HyClone, South Logan, UT, USA), penicillin (100 U/ml), and streptomycin (100 mg/ml).

### Wound healing and invasion assay

Wound healing and invasion assay were performed as described previously [13]. Images of SiHa and C33A cells migrating into the wound were captured at time points of 0 and 24 h by an inverted microscope (40×).

### Flow cytometry analyses

The ALDEFLUOR assay (STEMCELL Technologies, Vancouver, Canada) was performed as described previously [13]. As a negative control, an aliquot of ALDEFLUOR-exposed cells was immediately quenched with a specific ALDH inhibitor, diethylaminobenzaldehyde (DEAB). Following 30 min incubation at 37 °C, cells were washed and analyzed by FACSCalibur (Becton Dickinson, MD, USA).

### Spheroid formation assay

Wound healing and invasion assays were performed as described previously [13]. After 10 ~ 14 days, the number of SiHa and C33A cell spheroids (tight, spherical, non-adherent masses with >60 μm diameter) were counted, and spheres were imaged under an inverse microscope. Spheroid formation efficiency (%) = colonies/input cells × 100.

### Western blot analysis

We homogenized cell lysates in a lysis buffer (20 mM HEPES, 150 mM NaCl, 2 mM EGTA, 1 mM EDTA, 20 mM glycerol phosphate, 1 % Triton X-100, and 10 % glycerol with protease Sigma) and a phosphatase-inhibitor cocktail (Roche). Additional details were performed as described previously [14]. Membranes were primarily blotted with primary antibodies against GAPDH (AbFrontier); E-cadherin, β-catenin, vimentin, and Sox2 (Cell Signaling Technology); and OCT-3/4 (Abcam).

### Statistical analysis

Statistical analysis was performed using an ANOVA test and determination of the Pearson’s correlation coefficient on SigmaPlot 12.3 software. A *p* value of <0.05 was considered significant.

## Results

### A1E inhibited EMT in HPV 16-positive cervical carcinoma cells

**Fig. 1:**
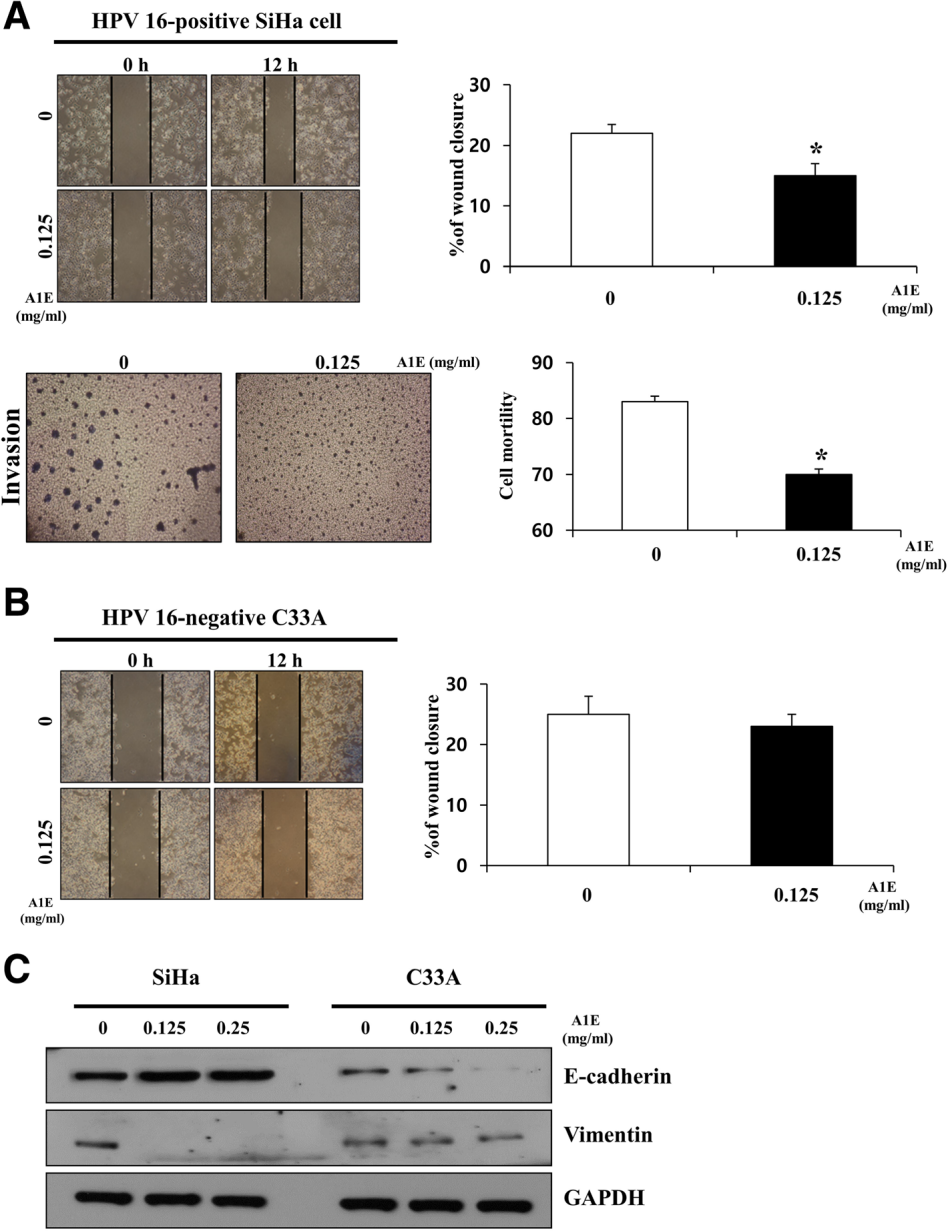
A1E reduces EMT in cervical carcinoma cells. **a** Wound healing and invasion assays of HPV 16-positive SiHa cells. **b** Wound healing assays of HPV-negative C33A cells. Data are presented as mean ± SEM. *P < 0.05. **c** Western blotting analysis of EMT markers in A1E-treated SiHa and C33A cells

Previously we demonstrated that A1E is cytotoxic against SiHa cells n vitro with an estimated significant effect on cell viability at 0.125 mg/ml [11]. Moreover, A1E perturbed cell cycle progression at the sub-G1 phase and altered cell cycle regulatory factors in SiHa cells. A1E activated apoptotic intrinsic pathway markers such as caspase-9, caspase-3, and poly (ADP-ribose) polymerase and down-regulated expression of Bcl-2 and Bcl-xl. A1E induced mitochondrial membrane potential collapse and cytochrome c release and inhibited phosphatidylinositol 3-kinase (PI3K)/Akt, which are key factors involved in cell survival signaling [11]. A1E exerts an anti-proliferative action, which inhibits the growth of cervical cancer cells through apoptosis, that demonstrates its anti-cervical cancer properties [15]. Based on these data, we characterized stemness-associated properties of HPV 16-positive SiHa and HPV 16-negative C33A cervical carcinoma cells and examined the effect of A1E on these properties. A1E targets E6 and E7 oncogenes; thus, A1E significantly inhibited proliferation of SiHa cells, whereas it did not affect proliferation of C33A cells that were only slightly affected by A1E [11]. This indicates very low expression levels of E6 and E7 oncogenes in C33A cells [16]. On examination of wound healing and invasion activities, we found that A1E reduced both activities in SiHa cells (Fig. 1a), but did not reduce wound healing activities in C33A cells (Fig. 1b). We next investigated whether A1E can regulate EMT, which is a major cause of tumor progression that causes cell migration and invasion, allows tumor metastasis, and establishes secondary tumors at distant sites [17]. A1E-treated HPV 16-positive SiHa cells showed significantly increased E-cadherin and decreased vimentin expression levels. However, A1E-treated HPV 16-negative C33A cells showed significantly decreased E-cadherin expression levels and unchanged vimentin expression levels (Fig. 1c). These data suggest that A1E negatively regulates EMT in HPV 16-positive SiHa cells.

### A1E reduced CSCs self-renewal in HPV 16-positive cervical carcinoma cells

**Fig. 2:**
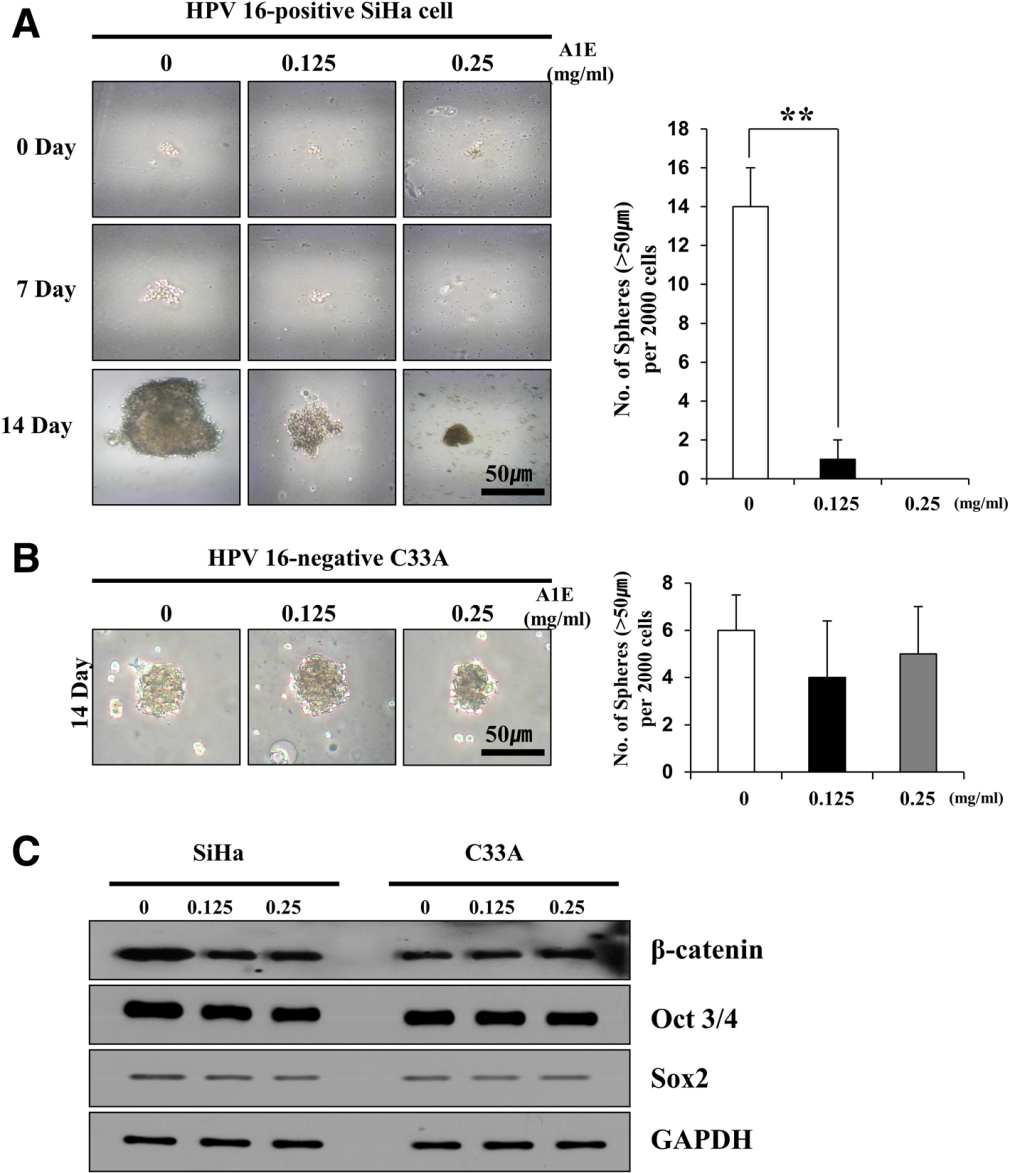
A1E regulates stemness in cervical carcinoma cells. **a** Spheroid formation assay of A1E-treated HPV 16-positive SiHa cells grown in stem cell selective media for 0, 7, and 14 days. **b** Spheroid formation assay of A1E-treated HPV-negative C33A cells grown in stem cell selective media for 14 days (magnification, 100×). Bar represents 50 μm. Histogram shows the average number of spheroids formed from 2000 cells. Data are presented as mean ± SEM. **P < 0.01. **c** Western blotting analysis of stemness markers in A1E-treated SiHa and C33A cells

Cultured CSCs can generate floating spheroid bodies [18]. Thus, we next investigated whether spheroid formation occurs in A1E-treated cervical cancer cells. In A1E-treated HPV 16-positive SiHa cell cultures, spheroid formation was observed within 0, 7, and 14 days, indicating that SiHa cells possess a significant proportion of CSCs (Fig. 2a). However, in A1E-treated HPV 16-negative C33A cells, regulation of spheroid formation (Fig. 2b) and stemness-related gene expression (Fig. 2c) was not observed. It has been reported that SiHa cells show significant expressions of stemness markers such as β-catenin and stemness-related genes such as Oct-3/4 and Sox2 [7]. Our data indicate that A1E-treated HPV 16-positive SiHa cells down-regulated sphere formation and β-catenin expression and slightly decreased stemness gene (OCT-3/4 and Sox2) expression (Fig. 2c). Taken together, A1E treatments inhibited spheroid formation and regulated OCT-3/4 and Sox2 expressions. These data suggest that A1E suppresses the self-renewal activity of the CSC subpopulation in HPV 16-positive SiHa cells.

### ALDH and CD 133 expression in A1E-treated cervical cancer cells

**Fig. 3:**
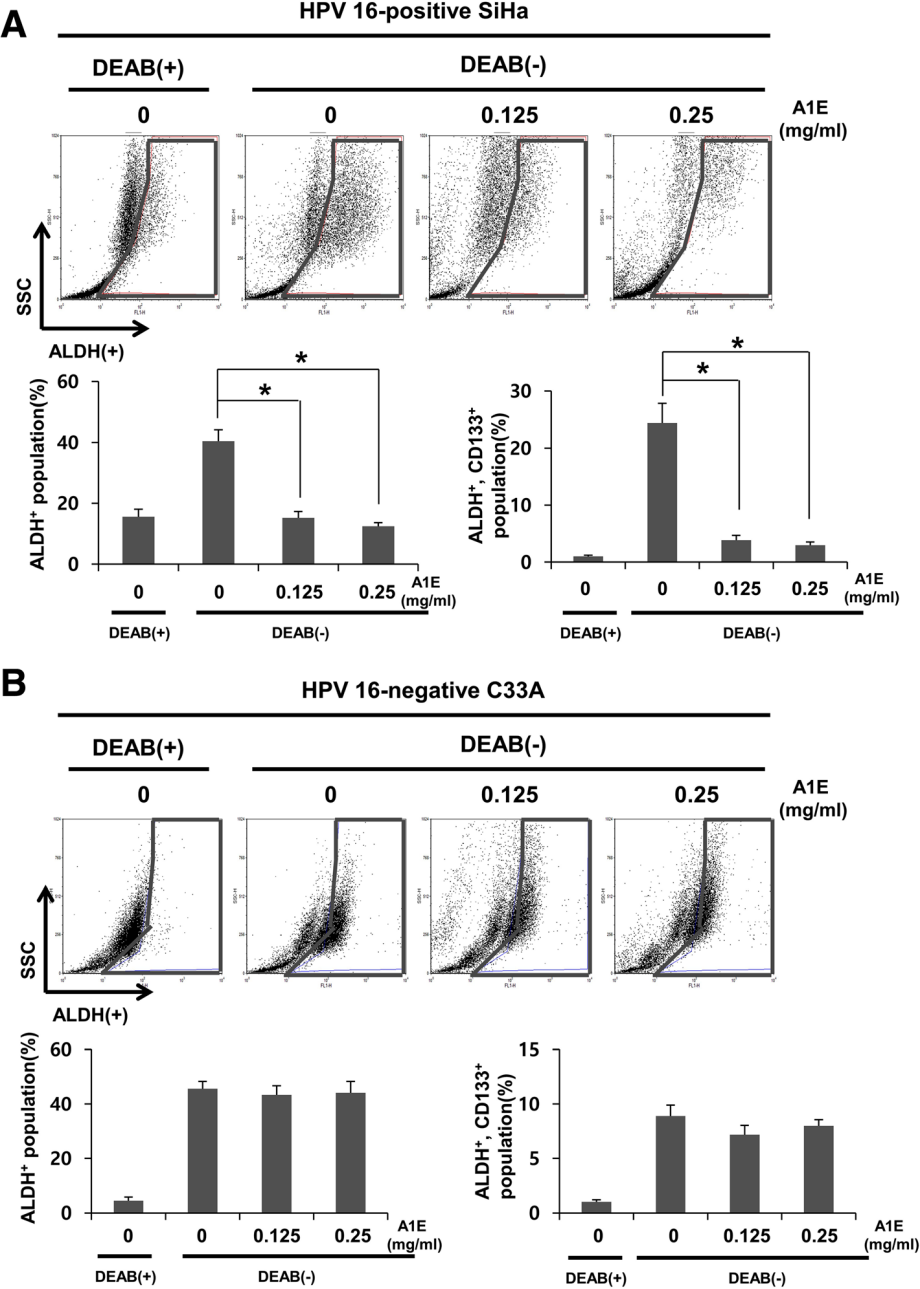
A1E regulates ALDH and CD133 positive expression in cervical carcinoma cells. **a** FACS analysis was performed on A1E-treated or untreated HPV 16-positive SiHa cells. **b** FACS analysis was performed on A1E-treated or untreated HPV-negative C33A cells. Data are presented as mean ± SEM. *P < 0.05

In recent reports, ALDH1 has been considered a marker for CSCs. ALDH activity has been used to isolate CSCs from cervical cancer cell lines and primary cervical cancer cells because ALDH+ cervical cancer cells possess self-renewal and differentiation abilities and have enhanced tumorigenicity [19]. The ALDEFLUOR kit was used to test ALDH enzymatic activity in HPV 16-positive SiHa and HPV 16-negative C33A cervical cancer cell lines. Cells were labeled with the activated ALDEFLUOR reagent in the presence or absence of the ALDH inhibitor, DEAB. A drop of ALDEFLUOR-labeled cells was examined by FACS Calibur [19]. We found that the numbers of ALDH+ and ALDH/CD133 double-positive cells were significantly decreased in A1E-treated SiHa cells than in untreated SiHa cells (Fig. 3a). Moreover, A1E treatment did not affect ALDH+ and ALDH/CD133 double-positive expression in HPV 16-negative C33A cells (Fig. 3b). These data indicate that A1E-treated SiHa cells have lower self-renewal capacity compared to untreated SiHa cells.

## Discussion

HPV is implicated in virtually all cervical cancers worldwide, with HPV 16 being the most common high-risk HPV type [2]. Since the discovery of the role of HPV in cervical cancer, many techniques have been developed to identify HPV infection and have been tested by screening patient groups [20]. It has been shown that results of HPV tests predict the risk of cervical cancer and its precursors better and sooner than examinations of cytological abnormalities [21]. Recent studies have revealed the critical role of CSCs in tumorigenicity and metastasis [4]. To date, CSCs have been identified in numerous solid cancers including breast and cervical cancers [19]. Moreover, CSCs are responsible for cancer recurrence. Based on these reports, further cancer therapies might focus on the elimination of CSCs [8]. Furthermore, development of innovative therapeutics for the treatment of cervical cancer is required.

Previously we assessed molecular mechanisms involved in anticancer effects of A1E [12]. A1E is composed of ethanol extracts from mixtures of ginseng, Pinellia tuber, Chaga, Alpinia rhizome, Sparganium rhizome, Alpiniakatsumadai seed, Arisaema rhizome, Psoraleae semen, cinnamon bark, and Dolichos seed [12]. A1E treatment significantly inhibited proliferation of SiHa cells, whereas it did not inhibit proliferation of CaSki, C33A, and HaCaT cells. Furthermore, we investigated whether A1E can regulate cervical CSC self-renewal in HPV 16-positive SiHa and HPV 16-negative C33A cervical carcinoma cells. However, A1E-treated SiHa showed decreased cell wound healing and invasion. Moreover, A1E-treated SiHa cells inhibited EMT markers such as E-cadherin and suppressed the expression of β-catenin. E-cadherin binds to β-catenin to improve cell–cell adhesion; thus, changes of E-cadherin and β-catenin expressions are correlated with tumor invasion and metastasis [22]. Additionally, A1E-treated SiHa cells showed downregulated spheroid formation and slightly decreased expression of stemness genes OCT-3/4 and Sox2. Notably, the aberrant expression of certain stem cell-related nuclear transcription factors, such as OCT-3/4 and Sox2, could contribute to cervical carcinogenesis. Sox2 could enhance the proliferation of cervical cancer cells by upregulating cyclin D1 expression [23]. We next investigated ALDH and CD133 activity in HPV 16-positive SiHa and HPV 16-negative C33A cells. ALDH1 and CD133 are cancer stem-cell markers and their presence strongly correlates with tumor malignancy and self-renewal properties of stem cells in cancers including cervical cancer [19]. Our data show that numbers of ALDH+ and ALDH/CD133 double positive cells were significantly decreased in A1E-treated SiHa cells. However, A1E treatment did not affect ALDH+ and ALDH/CD133 double positive expression and self–renewal capacity in HPV 16-negative C33A cells. Taken together, these data suggest that A1E extracts may be potentially used in therapies to suppress CSC self-renewal in HPV 16-positive SiHa cervical cancer cells.

## Conclusions

From the above findings, A1E is composed of 11 oriental medicinal herbs. A1E negatively regulates EMT in HPV 16-positive SiHa cells. Additionally, A1E-treated SiHa cells showed downregulated spheroid formation and slightly decreased expression of stemness genes OCT-3/4 and Sox2. Therefore, numbers of ALDH+ and ALDH/CD133 double positive cells were significantly decreased in A1E-treated SiHa cells. Thus, A1E reduces stemness and self-renewal in HPV 16-positive cervical CSCs.

## Abbreviations

ALDH: aldehyde dehydrogenase
CSC: cancer stem cell
DEAB: diethylaminobenzaldehyde
EMT: epithelial–mesenchymal transition
HPV: human papillomavirus

## References

[1] Bosch FX, Lorincz A, Munoz N, Meijer CJ, Shah KV (2002). The causal relation between human papillomavirus and cervical cancer. J Clin Pathol.

[2] Munagala R, Kausar H, Munjal C, Gupta RC (2011). Withaferin A induces p53-dependent apoptosis by repression of HPV oncogenes and upregulation of tumor suppressor proteins in human cervical cancer cells. Carcinogenesis.

[3] Martens JE, Arends J, Van der Linden PJ, De Boer BA, Helmerhorst TJ (2004). Cytokeratin 17 and p63 are markers of the HPV target cell, the cervical stem cell. Anticancer Res.

[4] Reya T, Morrison SJ, Clarke MF, Weissman IL (2001). Stem cells, cancer, and cancer stem cells. Nature.

[5] Lopez J, Poitevin A, Mendoza-Martinez V, Perez-Plasencia C, Garcia-Carranca A (2012). Cancer-initiating cells derived from established cervical cell lines exhibit stem-cell markers and increased radioresistance. BMC Cancer.

[6] Chhabra R: Cervical cancer stem cells: opportunities and challenges. J Cancer Res Clin Oncol. 2015;141(11):1889-97. 10.1007/s00432-014-1905-y PMC11823727 25563493

[7] Feng D, Peng C, Li C, Zhou Y, Li M, Ling B (2009). Identification and characterization of cancer stem-like cells from primary carcinoma of the cervix uteri. Oncol Rep.

[8] Vidal SJ, Rodriguez-Bravo V, Galsky M, Cordon-Cardo C, Domingo-Domenech J (2014). Targeting cancer stem cells to suppress acquired chemotherapy resistance. Oncogene.

[9] Mani SA, Guo W, Liao MJ, Eaton EN, Ayyanan A, Zhou AY (2008). The epithelial-mesenchymal transition generates cells with properties of stem cells. Cell.

[10] Lee JE, Seo I, Jeong SJ, Koh W, Jung JH, Kwon TR (2011). Herbal cocktail ka-mi-kae-kyuk-tang stimulates mouse bone marrow stem cell hematopoiesis and janus-activated kinase 2/signal transducer and activator of transcription 5 pathway. Am J Chin Med.

[11] Ham S, Bak Y, Kwon T, Kang JW, Choi KD, Han TY (2014). A1E Induces Apoptosis via Targeting HPV E6/E7 Oncogenes and Intrinsic Pathways in Cervical Cancer Cells. J Applied Biological Chemistry.

[12] Bak Y, Ham S, Baatartsogt O, Jung SH, Choi KD, Han TY (2013). A1E inhibits proliferation and induces apoptosis in NCI-H460 lung cancer cells via extrinsic and intrinsic pathways. Mol Biol Rep.

[13] Kim IG, Kim SY, Choi SI, Lee JH, Kim KC, Cho EW (2014). Fibulin-3-mediated inhibition of epithelial-to-mesenchymal transition and self-renewal of ALDH+ lung cancer stem cells through IGF1R signaling. Oncogene.

[14] Han YH, Kim SU, Kwon TH, Lee DS, Ha HL, Park DS (2012). Peroxiredoxin II is essential for preventing hemolytic anemia from oxidative stress through maintaining hemoglobin stability. Biochem Biophys Res Commun.

[15] Arrossi S, Sankaranarayanan R, Parkin DM (2003). Incidence and mortality of cervical cancer in Latin America. Salud Publica Mex.

[16] Wang-Johanning F, Lu DW, Wang Y, Johnson MR, Johanning GL (2002). Quantitation of human papillomavirus 16 E6 and E7 DNA and RNA in residual material from ThinPrep Papanicolaou tests using real-time polymerase chain reaction analysis. Cancer.

[17] Kupferman ME, Jiffar T, El-Naggar A, Yilmaz T, Zhou G, Xie T (2010). TrkB induces EMT and has a key role in invasion of head and neck squamous cell carcinoma. Oncogene.

[18] Lee J, Kotliarova S, Kotliarov Y, Li A, Su Q, Donin NM (2006). Tumor stem cells derived from glioblastomas cultured in bFGF and EGF more closely mirror the phenotype and genotype of primary tumors than do serum-cultured cell lines. Cancer Cell.

[19] Liu SY, Zheng PS (2013). High aldehyde dehydrogenase activity identifies cancer stem cells in human cervical cancer. Oncotarget.

[20] Walboomers JM, Jacobs MV, Manos MM, Bosch FX, Kummer JA, Shah KV (1999). Human papillomavirus is a necessary cause of invasive cervical cancer worldwide. J Pathol.

[21] Schiffman M, Wentzensen N, Wacholder S, Kinney W, Gage JC, Castle PE (2011). Human papillomavirus testing in the prevention of cervical cancer. J Natl Cancer Inst.

[22] Zhang H, Liu J, Yue D, Gao L, Wang D, Zhang H (2013). Clinical significance of E-cadherin, beta-catenin, vimentin and S100A4 expression in completely resected squamous cell lung carcinoma. J Clin Pathol.

[23] Ji J, Ning FR, Liu HJ, Wei X, Zhao J, Wang YL (2014). Effect of Sox2 on proliferation of cervical squamous cancer cell line SiHa. Sichuan Da Xue Xue Bao Yi Xue Ban.

